# Efficacy of ivermectin and albendazole combination in suppressing transmission of lymphatic filariasis following mass administration in Tanzania: a prospective cohort study

**DOI:** 10.1186/s40249-024-01214-3

**Published:** 2024-06-12

**Authors:** Adam M. Fimbo, Rajabu Hussein Mnkugwe, Eulambius Mathias Mlugu, Peter P. Kunambi, Alpha Malishee, Omary M.S. Minzi, Appolinary A. R. Kamuhabwa, Eleni Aklillu

**Affiliations:** 1 Department of Global Public Health, Karolinska Institutet at Karolinska University, Stockholm, Sweden; 2 Tanzania Medicines and Medical Devices Authority (TMDA), P. O, Box 77150, Dar Es Salaam, Tanzania; 3 Department of Clinical Pharmacology, School of Biomedical Sciences, Campus College of Medicine, Muhimbili University of Health and Allied Sciences, P. O, Box 65013, Dar Es Salaam, Tanzania; 4 Department of Pharmaceutics and Pharmacy Practice, School of Pharmacy, Muhimbili University of Health and Allied Sciences, P. O, Box 65013, Dar Es Salaam, Tanzania; 5 National Institute for Medical Research, P. O. Box 9653, Dar Es Salaam, Tanzania; 6 Department of Clinical Pharmacy and Pharmacology, School of Pharmacy, Muhimbili University of Health and Allied Sciences, P. O, Box 65013, Dar Es Salaam, Tanzania

**Keywords:** Circulating filarial antigen, Efficacy, Ivermectin, Albendazole, Lymphatic filariasis, Microfilariae, Mass drug administration, Tanzania

## Abstract

**Background:**

Preventive chemotherapy with ivermectin and albendazole (IA) in mass drug administration (MDA) programs for all at-risk populations is the core public health intervention to eliminate lymphatic filariasis (LF). Achieving this goal depends on drug effectiveness in reducing parasite reservoirs in the community to halt transmission. We assessed the efficacy of ivermectin and albendazole in clearing microfilariae and circulating filarial antigens (CFA) following MDA.

**Methods:**

This community-based prospective study was conducted in Mkinga district, Tanga region, Tanzania, from November 2018 to June 2019. A total of 4115 MDA-eligible individuals were screened for CFA using Filarial test strips. CFA positives were re-examined for microfilariae by microscopy. CFA and microfilariae positive individuals were enrolled and received IA through MDA campaign. The status of microfilariae and CFA was monitored before MDA, and on day 7 and six-month following MDA. The primary efficacy outcomes were the clearance rates of microfilariae on day 7 and six-months, and CFA at 6 months of post-MDA. The McNemar test assessed the proportions of microfilariae positive pre- and post-MDA, while Chi-square tests were utilized to examine factors associated with CFA status six months post-MDA.

**Results:**

Out of 4115 individuals screened, 239 (5.8%) tested positive for CFA, of whom 11 (4.6%) were also positive for microfilariae. Out of the ten microfilariae-positive individuals available for follow-up on day 7, nine tested negative, yielding a microfilariae clearance rate of 90% [95% confidence interval (*CI*): 59.6–98.2%]. Participants who tested negative for microfilariae on day 7 remained free of microfilariae six months after MDA. However, those who did not clear microfilariae on day-7 remained positive six-months post-MDA. The McNemar test revealed a significant improvement in microfilariae clearance on day 7 following MDA (*P* = 0.02). Out of 183 CFA-positive individuals who were available at 6-month follow-up, 160 (87.4%) remained CFA positive, while 23 became CFA negative. The CFA clearance rate at 6 months post-MDA was 12.6% (95% *CI*: 8.5–8.5%). There was no significant association of variability in ivermectin plasma exposure, measured by maximum concentration or area under the curve, and the clearance status of microfilariae or CFA post-MDA.

**Conclusions:**

Preventive chemotherapy with IA effectively clears microfilariae within a week. However, it is less effective in clearing CFA at six months of post-MDA. The low clearance rate for filarial antigenemia underscores the need for alternative drug combinations and additional preventive measures to achieve LF elimination by 2030.

## Background

Lymphatic filariasis (LF), is a painful and profoundly disfiguring neglected tropical disease that affects mostly deprived and poor communities in Africa, the Middle East, Central and South America, and Southeast Asia. Infection with *Wuchereria bancrofti* parasite is responsible for more than 90% of the global disease burden, which is endemic in 72 countries [1, 2]. Sub-Saharan Africa is the most affected continent globally in terms of the highest disease burden, with an estimated 251 million people living in areas with ongoing LF transmission [3]. The adult filarial worms primarily reside in the lymphatic vessels and lymph nodes, where they cause progressive damage and obstruction in the lymphatic system. The disease affects all age groups, but it is usually acquired during childhood, and its visible chronic manifestations, such as lymphoedema, scrotal swelling, and elephantiasis, may occur later in life [4, 5].

In 2000, the World Health Organization (WHO) launched a Global Programme to Eliminate Lymphatic Filariasis (GPELF) with the aim of achieving global elimination of the disease as a public health problem by 2020 [6], but recently the target milestone was extended to 2030 [7]. The World Health Organization (WHO) recommends implementing large-scale community-based mass drug administration (MDA) of anti-filarial drugs to all populations at risk of infection as the primary public health intervention to stop LF transmission in endemic regions [2]. The option of drug combination for MDA is based on onchocerciasis and loiasis co-endemicity to avoid the risk of serious adverse events. The recommended preventive chemotherapy consists of albendazole monotherapy in loiasis co-endemic areas, ivermectin with albendazole combination (IA) in onchocerciasis co-endemic, and or with diethylcarbamazine (DA), and non-endemic areas respectively [8]. Based on reports of superior efficacy, the WHO updated the guideline in 2017, recommending triple therapy with ivermectin, diethylcarbamazine and albendazole combination (IDA) in areas outside of Africa to accelerate the elimination of LF [2]. Although a 74% reduction in global infections since the start of GPLEF has been achieved, an estimated 51.4 million people were still infected in 2018 [9]. In 2021, about 882 million people in 44 countries lived in areas requiring preventive chemotherapy to halt transmission [8].

Tanzania is ranked as the third top African country in terms of highest LF prevalence and burden, with about 70% of the population at risk of infection and six million infected people living with debilitating manifestations of the disease [10, 11]. The prevalence of LF infection is higher in coastal areas along the Indian Ocean, including the Tanga region. Since 2002, the Tanzanian National Program for Elimination of Lymphatic Filariasis (NPELF) has implemented annual MDA with IA through a directly observed therapy. Although the MDA intervention significantly reduced LF transmission and morbidity [12–15], the target of eliminating it as a public health problem by 2020 was not achieved [16]. LF continues to afflict impoverished individuals, particularly those residing in rural areas and hotspots where signs of ongoing transmission persist. Despite efforts, 14 districts in Tanzania remained endemic to LF, necessitating continued MDA interventions as of 2020 [17].

Aligned with the global NTD roadmap [7], the updated Tanzania national NTD program targets to eliminate LF as a public health concern by 2030 [17]: defined as achieving a microfilaria prevalence of less than 1% in the at-risk population and confirming sustained infection rates below transmission assessment survey thresholds for a minimum of four years after cessation of MDA. Achievement of this target milestone relies on the drug's effectiveness in reducing parasite reservoirs in the community and interrupting transmission. While regular large-scale MDA has effectively decreased the disease burden, the continued exposure raises concerns about potential parasite tolerance and the emergence of drug resistance [18]. Detection of benzimidazole (albendazole) resistance-associated mutations in the filarial nematode *W. bancrofti* has been reported previously [19].

The Tanzanian National Neglected Tropical Diseases (NTD) program has underscored the importance of conducting post-MDA surveys and impact assessments. Several surveillance studies have evaluated the impact of multiple rounds of large-scale preventive chemotherapy with IA in Tanzania [12–15]. Despite a progressive decline in LF prevalence over a decade of MDA implementation, LF remains prevalent, and transmission persists in some districts, and the efficacy of IA on parasite clearance has not been systematically monitored. Drug efficacy can be influenced by various factors, including variations in drug exposure, genetic factors, the developmental stage of the parasite (microfilaria versus adult worms), and the potential for resistance [18]. Geographic disparities in drug efficacy have been documented, where 96% of treated individuals were free of microfilariae in Papua New Guinea [20] compared to 76% in Cote d'Ivoire after 12 months of therapy with triple therapy IDA [21]. This highlights the need for drug efficacy surveillance in various geographic locations and settings.

Assessing post-treatment changes in circulating filarial antigen (CFA) levels and microfilariae from nighttime blood smears can provide valuable insights into the macrofilaricidal (adult worm-killing) and microfilaricidal (larval stage-killing) effects of anti-filarial drugs, respectively [21–23]. Data regarding the parasitological efficacy of IA in infected individuals from Africa are limited, and the potential impact of variability in drug exposure on treatment outcomes remains largely unexplored. This study sought to address this gap by evaluating the effectiveness of routine MDA with IA in eliminating CFA and microfilaremia, thereby reducing the parasite reservoir within the community and interrupting transmission. Therefore, we investigated the efficacy and associated factors of a single dose of IA administered as preventive chemotherapy during MDA, in terms of clearing microfilariae and antigenemia, among individuals infected with LF residing in a rural endemic district of Tanzania.

## Methods

### Ethical statement

Ethical clearance was granted by the Medical Research Coordinating Committee of the National Institute for Medical Research, Tanzania (NIMR/HQ/R.8a/Vol. IX/2890). Meetings with district authorities and village leaders were conducted to obtain permission to conduct research activities in the community. Sensitization meetings were held in each village to explain the objectives and methodology of the study as well as to seek community consent. Questions were asked, and the study team provided explanations during these meetings. Informed consent and/or assent were obtained both orally and in writing from all individual participants and/or parents/guardians in the case of children. Confidentiality was maintained during and after the study.

### Study area

**Fig. 1:**
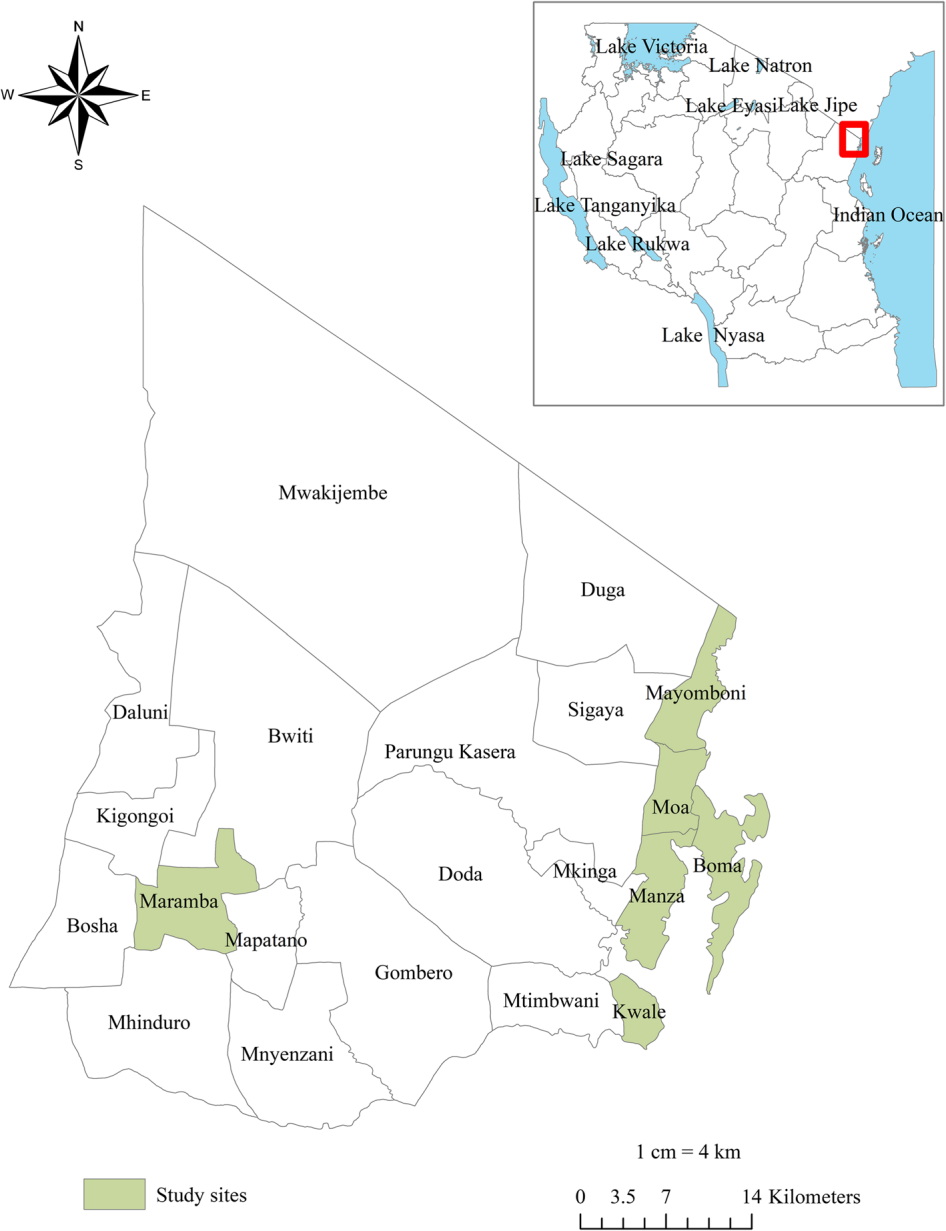
Map of the study site. In the top left corner, there is a map of Tanzania, situated in the eastern part of Africa. The red square highlights the Tanga region, where Mkinga district is located. The bottom left section displays a detailed map of the wards within the Mkinga district, indicating the villages that participated in this study. This study site map was created using ArcGIS software version 10.7.1 (Esri, California, USA)

The study was conducted in LF endemic communities of the Mkinga district, Tanga region, which is a target area for MDA by the Neglected Tropical Diseases Control Program (NTDCP) in Tanzania. The district is bordered by Muheza district and Tanga city to the South, the Indian Ocean to the East, Korogwe and Lushoto districts to the West and the Kenya border to the North. The district has two main rainy seasons annually (bimodal), the long rains from March to June and the less intensive short rains from November to December. Tanga is a warm and wet climate region with no significant variation of temperature at the coast due to the influence of the Indian Ocean. Humidity is high and often goes up to 100% maximum ranging from 65% to 70% minimum. There are health facilities in most villages in the district, and most of the population has access to a health facility within six kilometers. According to the national census conducted in 2022, the Mkinga district population was 146,802, with 49.8% (73,048) being males (14). The altitude ranges from 0 to 1506 m above sea level measured from the Nilo peak (15). The main economic activities in this district include fishing, subsistence farming, low-scale livestock keeping, and petty trading for the rest. Figure 1 depicts the location of the study sites.

### Study design and population

This prospective longitudinal efficacy study was nested in a larger community-based cross-sectional study conducted between November 2018 and January 2019 [16]. In brief, two weeks prior to MDA, a total of 4115 MDA-eligible individuals (49.7% males, 35.2% children) were screened for CFA across 15 villages. Those testing positive for CFA were further screened for microfilaremia. All eligible residents, regardless of their CFA or microfilariae status, received MDA following the WHO guidelines [2, 24]. All individuals who tested positive for CFA, including those who also tested positive for microfilariae before MDA, were enrolled and monitored for clearance of CFA and microfilariae following MDA. Pregnant women and children under five years old were excluded from the study due to contraindications for the medications used.

### Treatment and follow-up

In Tanzania, residents in LF- endemic districts are eligible to receive annual MDA comprising IA combinations as preventive chemotherapy, without prior diagnosis following the WHO and national MDA program guidelines [2, 24]. Accordingly, all residents in our study area, including those who tested positive for CFA and microfilariae in our study, received single dose IA combinations as preventive chemotherapy through MDA campaign. The MDA was implemented by the National NTD control program through house-to-house visits, and the study team had no role in the planning or administration of the drugs. During the MDA campaign, residents were advised to take the medicine after food intake. Food intake was not considered in the study as it was observational and followed routine protocols established by the national MDA program.

After recording baseline sociodemographic, clinical, and medical history, including any comorbidities and concomitant medications, participants received a standard dose of ivermectin based on height (corresponding to 150–200 μg/kg) and albendazole 400 mg as recommended by the WHO [2, 24]. Ivermectin tablets were donated by Merck Sharpe and Dohme (MSD), Haarlem, The Netherlands. Albendazole was donated from GlaxoSmithKline (GSK), Brentford, UK.

On MDA Day, all MDA-eligible residents of the community, including those who were screened the diseases and those tested positive for CFA and microfilariae received a standard dose of IA as preventive chemotherapy under direct observed therapy. A longitudinal follow-up was conducted on a cohort of enrolled individuals who initially tested positive for CFA and microfilariae prior to MDA to monitor the changes in microfilariae and CFA positivity status after MDA. The clearance of microfilariae was assessed at both seven days and six months post-MDA, while the clearance of CFA was specifically evaluated at the six-month mark following MDA.

### Assessment of circulating filarial antigenemia

All individuals who tested positive for CFA before MDA underwent re-examination six months after receiving MDA to assess the efficacy of IA in clearing antigenemia. In brief, CFA detection test was performed on finger prick blood samples collected from study individuals using the rapid test for circulating filarial antigenemia (Filariasis Test Strip or FTS; Alere, Inc) as described previously [16]. Briefly, approximately 75 µl of blood was applied on Alere™ FTS (Alere©, Waltham, United States) using a special pipette with a 75 µl mark, and results were read at 10 min and recorded as CFA positive and negative. This test was done before MDA and was repeated at six months following MDA. FTS-positive tests were read by two independent study team members, and positives were retested.

### Assessment of microfilaraemia

All CFA-positive individuals were subsequently re-examined for microfilaraemia before and after receiving MDA using night blood smear microscopy. In brief, night blood sample collection from CFA-positive individuals was conducted between 21:00 and 1:00 to maximize the detection of microfilariae in the blood [25]. Finger prick blood samples were collected into 75 μl capillary tubes and transferred into a 1.8 ml cryotube containing 900 µl of 3% acetic acid, mixed thoroughly and transferred into Sedgwick-Rafter counting chamber. LF parasitemia was estimated by counting the number of microfilariae in the chamber using a compound light microscope set at 4 × magnification located at the National Institute for Medical Research (NIMR) laboratory in Tanga. Results were reported as number of microfilariae per 75 µl of blood. To ensure quality control, the readings for microfilariae for each night blood sample were done by two well-trained and experienced laboratory technicians, who discussed and agreed on the results. All positive tests were also reviewed by the study leader or designated scientist. Blood sampling was done at a village meeting point (health facility or village office building) in most cases. The door-to-door approach was also used under special circumstances, especially when individuals did not show up for blood sampling.

### Determination of ivermectin plasma concentrations

Two milliliters of venous blood were drawn at 0, 2, 4, and 6 h post-drug administration from the antecubital arm vein. The plasma levels of ivermectin were quantified using liquid-chromatography tandem mass spectrometry [26]. Population pharmacokinetic (PopPK) modeling of data was done using NONlinear Mixed Effects Modeling (NONMEM) as described previously [27]. Non-compartmental analysis (NCA) with linear trapezoidal rule was used to calculate maximum drug concentration (Cmax) in ng/ml and area under the curve (AUC_0–6 h_), in ng·h·ml^−1^. The Cmax and AUC were assessed for between-subject variability (coefficient of variation—CV%). Association of variability in ivermectin pharmacokinetic parameters with clearance of microfilariae and CFA after MDA was analyzed.

### Data management and statistical analysis

Data was collected electronically using tablets and submitted to the central server daily at the National Institute for Medical Research, Tanga Medical Research Centre laboratory. The Open-source data kit (ODK, https://opendatakit.org/) software was used to create the database and data collection applications. The data manager ensured daily precision and consistency checks, resolving queries promptly. Continuous data cleaning and validation were conducted, with periodic reports generated. Descriptive statistics summarized sociodemographic and clinical characteristics. To assess the impact of MDA, the McNemar test was employed to compare the proportions of microfilarial positives before and after the intervention. Factors associated with CFA status at six months were analyzed using either the Chi-square or Fisher’s exact tests. Univariable and multivariable logistic regression analyses were conducted to control for potential confounders. Factors with a *P*-value of < 0.2 in the univariable analysis or with clinical relevance and previously reported associations were included in the multivariable model. All *P*-values in statistical tests were two-sided, with a significance level set at < 0.05. Data visualization and analysis were performed using GraphPad Prism version 10 (GraphPad Software, San Diego, California, USA).

## Results

### Study enrollment, follow up and outcome

**Fig. 2:**
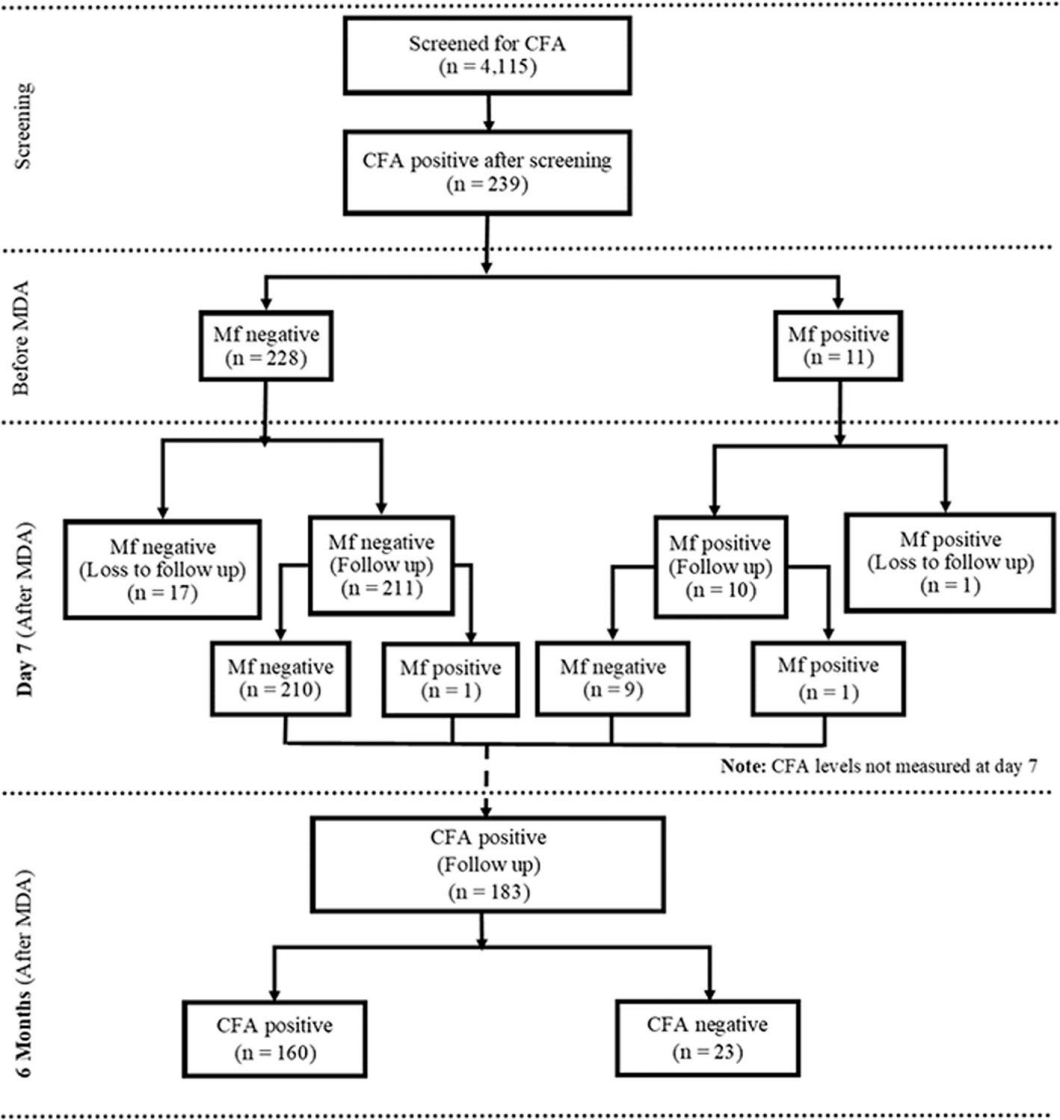
Study flow chart illustrating the screening, enrollment, and follow-up of study participants to monitor the clearance rate of microfilariae (mf) and circulating filarial antigens (CFA) on day seven and six months after receiving mass drug administration (MDA)

Before MDA, 239 individuals, constituting 5.8% of the screened population, tested positive for CFA. Among them, 11 individuals were also positive for microfilariae. All these individuals were enrolled and followed to assess the clearance rates of microfilariae and CFA following MDA. Microfilariae clearance was evaluated at seven days and six months post-MDA, while CFA clearance was assessed at six months post-MDA. The primary study outcomes were the proportion of participants free from microfilariae at day 7 and six months of post-MDA, as well as those free from filarial antigenemia at six months post-MDA. Figure 2 shows the study flow chart including screening, enrollment and status of microfilariae and CFA positivity during the study follow up.

### Sociodemographic and clinical characteristics

The median age of individuals was 34 years (IQR: 22–55 years), with males being higher than females (71.4%). The body mass index (BMI) was normal for most individuals (59.1%). A substantial number of individuals were using bed nets (89.5%) and only 8.0% reported practicing indoor residual spraying. Individuals who took part in the last MDA were less than half (42.6%) of the study population. Other sociodemographic and clinical characteristics are summarized in Table 1.

**Table 1:** Sociodemographic and clinical characteristics of the study participants at enrolment

Variables		Frequency (*n*)	Percent (%)
Sex	Male	170	71.4
	Female	68	28.6
BMI category (*n* = 235)	Underweight (< 18.5)	60	25.5
	Normal (18.5–24.9)	139	59.1
	Overweight (25.0–29.9)	29	12.3
	Obese (≥ 30.0)	7	3.0
Area of residence (ward)	Boma	13	5.5
	Manza	17	7.1
	Maramba	20	8.4
	Mayomboni	19	8.0
	Moa	21	8.8
	Kwale	148	62.2
Last menstrual period (*n* = 58)	Definitely < 4 weeks ago	22	37.9
	Post-menopause	22	37.9
	Weeks or longer	7	12.1
	Uncertain	7	12.1
Comorbidities (*n* = 13)	Hypertension	5	38.5
	Asthma or chronic lung disease	1	7.7
	Diabetes	2	15.4
	HIV	1	7.7
	Others	4	30.8
Median age in years (IQR)		34 (22, 55)	
Use of bed net		213	89.5
House windows screen		74	31.1
Indoor spraying		19	8.0
LF manifestations (*n* = 235)^a^		22	9.4
Concomitant medications (*n* = 235)		5	2.1
Took part in last MDA (*n* = 235)		100	42.6
Ever used ivermectin (*n* = 235)		75	31.9
Ever used albendazole (*n* = 235)		85	36.2

^a^LF manifestations – hydrocele, chylocele, adenolymphangitis and swelling of arms and limbs

*BMI* Body mass index, *LF* Lymphatic filariasis, *MDA* Mass drug administration

### Effects of treatment on microfilaremia clearance

**Fig. 3:**
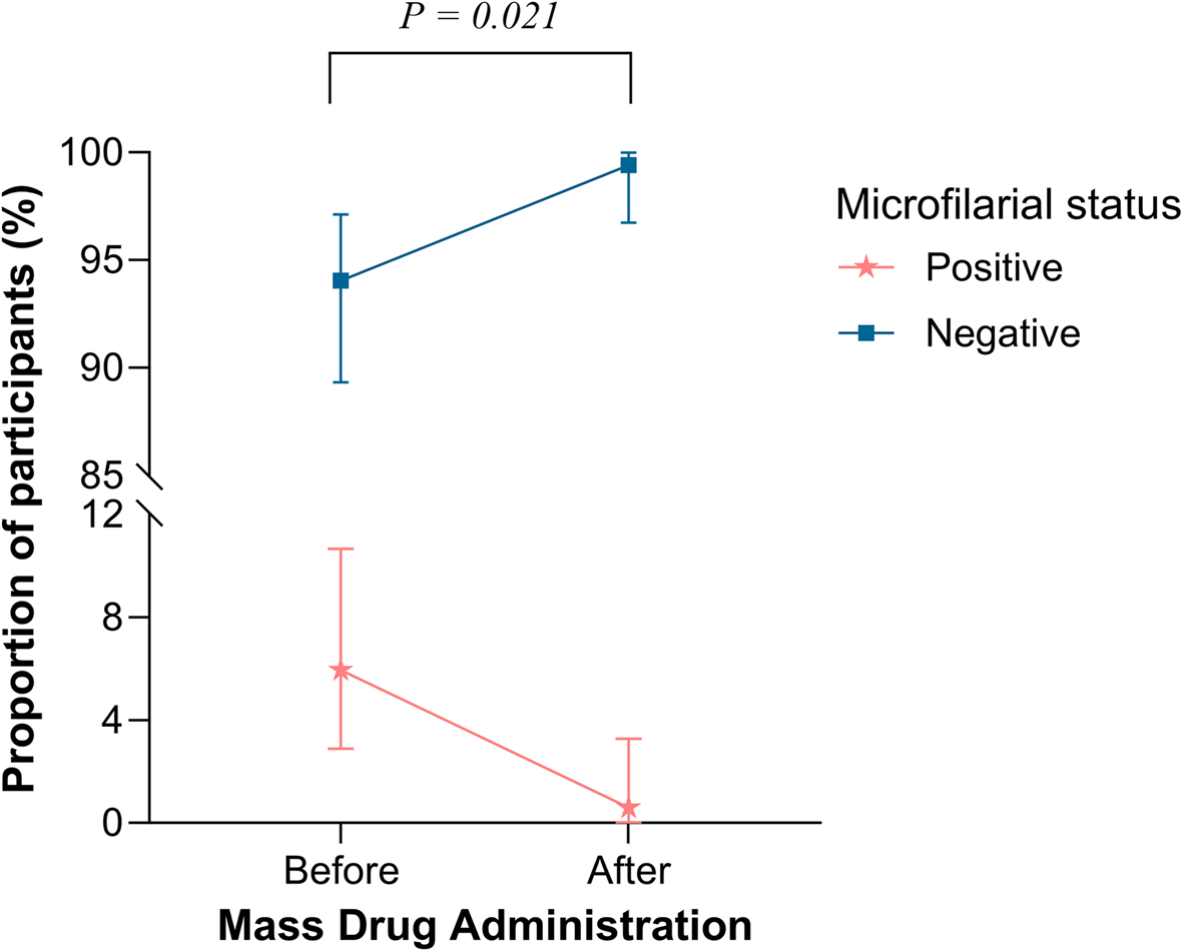
Interval plot of microfilarial clearance on day 7 post-mass drug administration (MDA). The bars represent the 95% confidence intervals for the proportions

Before the implementation of MDA, 239 individuals tested positive for CFA, among whom 11 individuals (5.8%) were also positive for microfilariae; four individuals had a parasite count of 2 per 75 µl of blood, while the remaining had 3, 5, 11, 15, 24, 28, and 61 parasites per 75 µl of blood. The median (interquartile range, IQR) was 5 parasites (2–24) per 75 µl of blood. Out of the 11 individuals who were microfilariae positive at baseline, ten were available for a follow-up test on day 7 of receiving MDA. Among these ten individuals, 9 (90%) tested negative for microfilariae on day 7 post-MDA. Nonetheless, this one individual showed a decrease in parasite count from 28 to 13 microfilariae/ml on day 7. Additionally, out of those who were microfilariae negative at baseline, one individual (0.6%) was tested microfilariae positive on day 7. The McNemar test indicated a significant change in microfilariae status before and after MDA (see Fig. 3).

At the six-month follow-up test, a total of 168 individuals were available for night blood sampling for microfilariae microscopy. Among those who were microfilariae-free on day 7, including the nine individuals who had cleared microfilariae, remained microfilariae-negative at the six-month of post-MDA. Conversely, the two individuals who tested positive for microfilariae on day 7 remained microfilariae-positive after six months of post-MDA.

### Microfilariae clearance and ivermectin plasma exposure

**Fig. 4:**
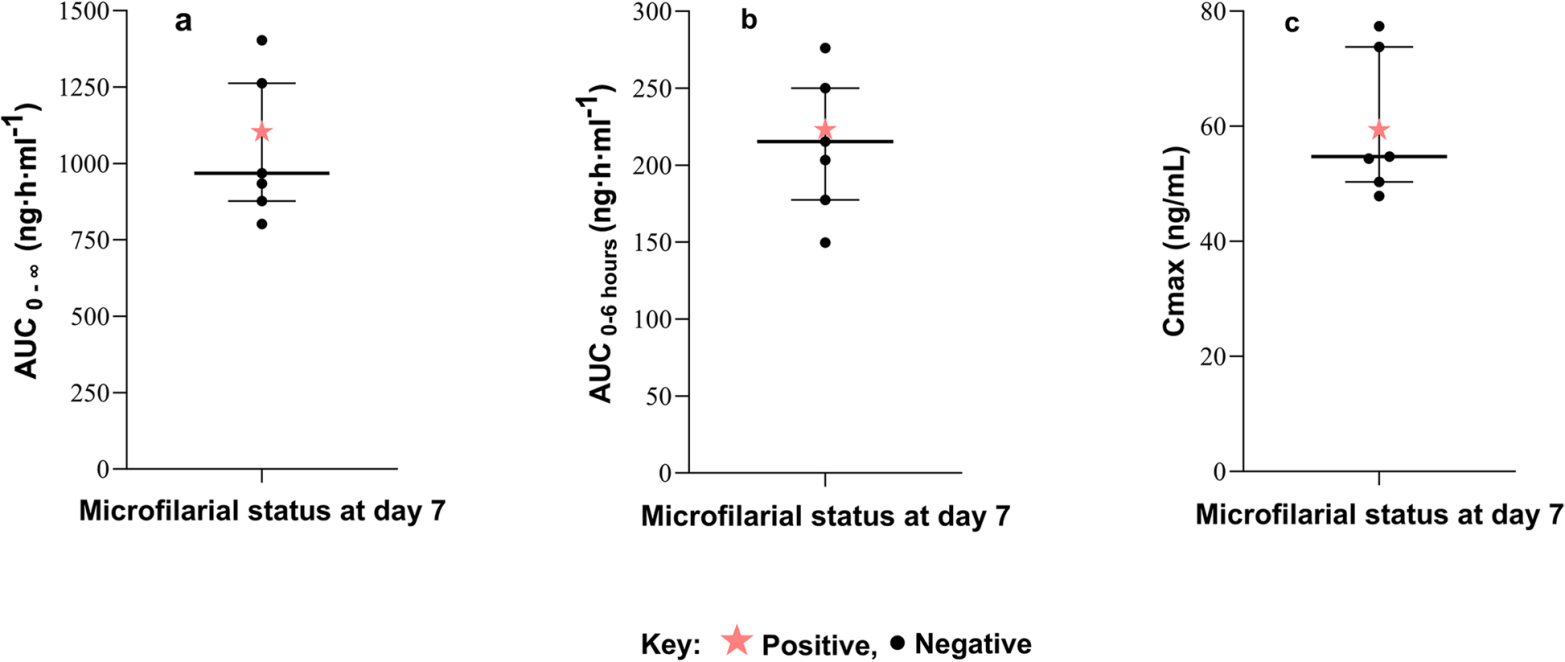
Dot plot showing plasma exposure of ivermectin among microfilariae-positive participants, categorized by their status (positive or negative) on day 7 post-MDA. The median and interquartile range are indicated. *AUC* Area under the curve, *Cmax* Maximum concentration

Individuals who cleared microfilariae on day 7 had minimal inter-individual variability in drug exposure as measured by Cmax and AUC. Figure 4 depicts that the Cmax of ivermectin ranged between 47.9 and 77.4 ng/ml (CV = 19.2%) while AUC _0-∞_ and AUC _0–6 h_ were 802.3 to 1402.8 ng·h·ml^−1^ (CV = 20.7%) and 149.77 to 276.01 ng·h·ml^−1^ (CV = 19.9%), respectively. The individual who did not clear microfilariae on day 7 had a comparable plasma drug exposure (Fig. 4).

### Effects of treatment on circulating filarial antigenemia clearance

Out of the initial cohort of 239 individuals who tested positive for CFA before receiving MDA, a follow-up was conducted six months after MDA for those available (*n* = 183). Within this follow-up group, only 23 (12.6%) individuals demonstrated a conversion from CFA positive to CFA negative status. The majority of participants (87.4%) remained CFA positive after six months of receiving MDA with IA. Six months following the MDA, the clearance rate of CFA was observed to be 12.6% (95% *CI*: 8.52 to 18.5%).

### Factors associated with circulating filarial antigenemia clearance

Factors associated with CFA status after 6 months following MDA were determined using Chi-square or Fishers exact tests (Table 2). Area of residence (ward) was the only significant factor associated with CFA status, whereby residents of Maramba ward significantly cleared CFA levels (37.5%) compared to other wards (*P* = 0.024). All other factors were not significantly associated with CFA status.

**Table 2:** Factors associated with circulating filarial antigenemia (CFA) clearance status six months after mass drug administration

Variable		CFA status six months post-MDA		*P*-value
		**Positive** ***n* (%)**	**Negative** ***n* (%)**	
Sex	Male	112 (86.2)	18 (13.8)	0.41
	Female	48 (90.6)	5 (9.4)	
Comorbidities	Yes	12 (100.0)	0 (0.0)	0.17
	No	146 (86.4)	23 (13.6)	
Use of bed net	Yes	144 (87.8)	20 (12.2)	0.71
	No	16 (84.2)	3 (15.8)	
House window screen	Yes	49 (87.5)	7 (12.5)	0.98
	No	111 (87.4)	16 (12.6)	
Indoor spraying	Yes	12 (92.3)	1 (7.7)	1.00
	No	148 (87.1)	22 (12.9)	
Took part in last MDA	Yes	72 (87.8)	10 (12.2)	0.85
	No	86 (86.9)	13 (13.1)	
BMI category	Underweight (< 18.5)	41 (85.4)	7 (14.6)	0.82
	Normal weight (18.5–24.9)	91 (88.3)	12 (11.7)	
	Overweight and Obese (≥ 25)	26 (86.7)	4 (13.3)	
LF manifestations	Yes	14 (77.8)	4 (22.2)	0.25
	No	144 (88.3)	19 (11.7)	
Area of residence (ward name)	Boma	9 (100.0)	0 (0.0)	0.02
	Manza	13 (92.9)	1 (7.1)	
	Maramba	10 (62.5)	6 (37.5)	
	Mayomboni	16 (100.0)	0 (0.0)	
	Mao	11 (78.6)	3 (21.4)	
	Kwale	101 (88.6)	13 (11.4)	

*CFA* Circulating filarial antigen, *BMI* Body mass index, *LF* Lymphatic filariasis, *MDA* Mass drug administration

There were no significant differences in the geometric mean of ivermectin Cmax (*P* = 0.19) and AUC (*P* = 0.41) between CFA-positive and negative individuals at six months of receiving IA. The univariable and multivariable analyses revealed that none of the factors tested were significantly associated with CFA clearance at six months following MDA (*P* > 0.05) (Table 3). Factors included in the multivariable model were based on clinical relevance and previously reported associations.

**Table 3:** Univariable and multivariable analysis of factors associated with circulating filarial antigen clearance

		**Univariable analysis**			**Multivariable analysis**		
**Variable**	**Category**	**c*OR***	**95% *CI***	***P*-value**	**a*OR***	**95% *CI***	***P*-value**
Age in years		0.99	0.96–1.01	0.21	0.98	0.96–1.01	0.13
Sex	Male	1.54	0.54–4.40	0.42	1.35	0.45–4.01	0.59
	Female	1^a^					
Use of bed net	Yes	0.74	0.20–2.77	0.66			
	No	1^a^					
House window screen	Yes	0.99	0.38–2.56	0.98			
	No	1^a^					
Indoor spraying	Yes	0.56	0.07–4.53	0.59			
	No	1^a^					
Took part in last MDA	Yes	0.92	0.38–2.22	0.85	1.06	0.43–2.61	0.89
	No	1^a^					
BMI category	Underweight	1.30	0.48–3.53	0.61			
	Over & Obese	1.17	0.35–3.92	0.80			
	Normal	1^a^					
LF manifestations	Yes	2.17	0.65–7.26	0.211	2.52	0.69–9.26	0.16
	No	1^a^					

*cOR* crude odds ratio, *aOR* adjusted odds ratio,* 1*^*a*^ Reference category. *BMI* Body mass index, *LF* Lymphatic filariasis, *MDA* Mass drug administration

## Discussion

We recently reported that after multiple rounds of MDA with IA, the infection prevalence in our study district did not decrease beyond levels where recrudescence is unlikely to occur [16]. Given the persistent prevalence of LF in the study area, drug efficacy studies are essential to determine whether the levels of microfilaremia and antigenemia are declining after MDA. In the current study, we prospectively monitored the clearance rate of filarial antigenemia and microfilaremia in LF-infected individuals seven days and six months after MDA. Our findings indicate that IA administered as preventive chemotherapy effectively clears microfilaremia (90%) from the blood within a week of drug administration with no rebound after six months. However, IA is less effective (12.6%) in clearing circulating filarial antigenemia six months post-MDA. To our knowledge, this is the first prospective study to assess the efficacy of IA in clearing microfilaremia and circulating filarial antigenemia among LF-infected individuals and its correlation with the variability in ivermectin drug exposure in Tanzania.

The high microfilariae clearance rate on day seven and the absence of microfilariae six months post-MDA in our study align with previous reports of rapid clearance of circulating microfilariae within the first five days [28]. A systematic review reported 98% to 100% microfilariae loss and 83% to 100% worm productivity loss following IA treatment [29]. Our study found no rebound microfilaremia up to six months post-MDA. This suggests that the treatment influenced the survival and production of microfilariae rather than affecting the survival of adult worms. Available evidence indicates that IA is safe [30] and effective in killing microfilariae and suppressing microfilariae production by temporarily sterilizing the long-lived adult worms, but the treatment does not kill the adult worm [29, 31]. Despite the success of repeated MDA in reducing the disease burden and transmission, LF persists in many countries, including Tanzania, partly due to the limited efficacy of IA against adult filarial worms [32].

Circulating filarial antigen serves as a surrogate marker of filarial infection, expressed by viable adult worms [33, 34]. Hence, macrofilaricidal activity of anti-filarial drugs can be indirectly evaluated by post-treatment changes in CFA levels [22]. In our study, only 12.6% of the LF-infected individuals were free from CFA after six months of receiving MDA. This finding signifies that IA has less effect on CFA levels post-MDA. Similar studies have reported an insignificant impact of IA on antigenemia clearance [21]. The existence of CFA indicates that an individual had acquired infection earlier in life as microfilariae takes time to mature into adult worms.

Available reports indicate that IA targets the microfilariae stage of filarial worms and does not exhibit a macrofilaricidal effect. Profound suppression of microfilaraemia but no change in movements characteristic of the adult worm on ultrasound examinations have been reported after six months of high dose ivermectin therapy [35, 36]. Hence, an alternative drug combination more effective in targeting adult worms, such as diethylcarbamazine, which exhibits both microfilaricidal activity and efficacy against adult worms, is warranted. Studies have demonstrated that diethylcarbamazine has a more microfilaricidal effect than ivermectin, significantly reducing CFA levels after treatment compared to ivermectin [22]. Superior efficacy of triple therapy with IDA compared to dual therapy with DA or IA has been reported [20, 21, 37]. Treatment with a single dose IDA resulted in sustained clearance of microfilariae in 96% of individuals with moderate to heavy infections of *W. bancrofti* for up to 3 years [20, 37]. In 2017, the WHO endorsed the use of a triple regimen with IDA in MDA programs in areas outside of Africa to accelerate the control and elimination of LF [2]. However, the deployment of triple therapy in Africa faces obstacles due to the potential for serious adverse effects of diethylcarbamazine in onchocerciasis patients or individuals infected with *Loa loa*, thereby limiting its usage across much of the continent [2, 37]. The use of diethylcarbamazine in such areas may result in serious adverse events, including the Mazzotti reaction, characterized by fever, swollen tender lymph nodes, tachycardia, and hypotension, which can be fatal [38]. Kenya, a country where LF is not co-endemic with onchocerciasis or loiasis, was the first African country to pilot MDA with IDA in 2018. Following this, Kenya reported the safety and tolerability of single-dose therapy with IDA compared to the standard DA regimen [39]. The co-endemicity of LF with onchocerciasis in Tanzania impedes the use of IDA due to potential adverse effects associated with diethylcarbamazine in patients with onchocerciasis. This underscores the complexity of managing NTDs in areas where multiple diseases overlap. Recently, there has been a call for re-evaluating co-endemic regions in sub-Saharan Africa and adjusting MDA protocols through implementing "test & treat" strategies adapted to the type and level of co-endemicity and preventing severe adverse events [3]. Considering that there are regions where either LF or onchocerciasis exists as mono-endemic [3], we recommend further investigations to re-map the co-endemicity of LF and onchocerciasis for targeted interventions with IDA in areas where onchocerciasis is not co-endemic. This approach will facilitate the elimination of LF as a public health problem by 2030 in Tanzania.

The present study explored any correlation between drug efficacy and variability in ivermectin plasma exposure levels using population pharmacokinetic (PK) parameters (Cmax and AUC) [27]. Our results indicate no significant correlation of variability in ivermectin plasma exposure with microfilariae and CFA clearance status. Indeed, recent population PK studies, including ours, indicated that the PK parameters of ivermectin were unaffected by LF infection status [27, 40]. Minimum inter-individual variability was observed in our study, as measured by the coefficient of variation (CV). The CV values were within the acceptable range (≤ 20%), indicating that all individuals had comparable exposure. To our knowledge, no studies have reported the relationship between IA exposure and microfilariae clearance. Therefore, the demonstration of the relationship between drug exposure and microfilariae clearance is the strength of our study.

A notable reduction in antigenemia was observed in Maramba ward, which is recognized as a hotspot for LF surveillance within Mkinga district [16]. Maramba village has been the focal point for LF interventions under the NTD program, encompassing various initiatives such as community sensitization campaigns, educational programs, anthropological studies, and sentinel and spot-check surveys. This comprehensive approach to disease control and surveillance have likely contributed to the observed decline in antigenemia in Maramba.

Despite the strengths, our study had also limitations. While other randomized clinical trials followed their study participants for durations spanning from 6 to 24 months post-MDA [21, 37], the follow-up period in our study was restricted to six months, which can be considered as a study limitation. Another limitation of our study was that despite initially enrolling 239 participants from 15 rural villages, only 183 were available for the six-month follow-up, resulting in a loss to follow-up rate of 23%. This occurred partly due to the nocturnal periodicity of microfilariae [25], which necessitated participants to stay overnight at the health center for night blood withdrawal and subsequent microscopic examination. Due to resources limitations in remote rural settings, we were unable to conduct a quantitative CFA assay, and this may be considered as study limitation. Instead, we used Filariasis Test Strip, a practical tool recommended by the WHO for routine use in LF elimination programs. CFA positivity identified through this method serves as an indicator of filarial infection, reflecting the presence of viable adult worms [33, 34]. Nevertheless, despite these limitations, our study stands out for its significant sample size in prospectively monitoring the effectiveness of IA in clearing microfilariae and circulating filarial antigen at a community level. We believe that our findings have significant implications for the ongoing efforts to control and eliminate LF, particularly in resource-constrained settings.

## Conclusions

The results of this study confirm the effectiveness of ivermectin-albendazole preventive chemotherapy against the larval stage of *W. bancrofti* in infected individuals, thereby effectively reducing parasite reservoirs and transmission in endemic communities. The use of IA combination in MDA campaigns rapidly and efficiently clears microfilariae within a week, with no rebound observed after six months of therapy. However, IA demonstrates reduced efficacy in clearing circulating filarial antigen six months post-treatment. Hence, repeated MDA rounds are imperative to effectively interrupt disease transmission in endemic regions. Tanzania aims to control and eliminate LF as a public health problem by 2030. To expedite progress toward this goal, we recommend re-mapping of LF and onchocerciasis co-endemicity in Tanzania to facilitate the inclusion of diethylcarbamazine in MDA programs and the adoption of triple therapy (IDA) in LF mono-endemic areas. Moreover, in regions where onchocerciasis is co-endemic, it is advisable to consider implementing bi-annual MDA with IA, especially in areas with high LF transmission rates.

## Data Availability

All data generated or analyzed during this study are included in the manuscript.

## Abbreviations

AUC: Area under the curve
BMI: The body mass index
Cmax: Maximum drug concentration
*CI*: Confidence interval
GPELF: Global Programme to Eliminate Lymphatic Filariasis
IA: Ivermectin with albendazole combination
IDA: Tiple therapy with ivermectin, diethylcarbamazine and albendazole combination
LF: Lymphatic filariasis
MDA: Mass Drug Administration
NTD: Neglected Tropical Diseases
SSA: Sub-Saharan Africa
WHO: World Health Organization
